# The Empowering Example of High Vaccine Uptake among the Arab Minority in Israel

**DOI:** 10.1093/eurpub/ckac129.105

**Published:** 2022-10-25

**Authors:** J Essa-Hadad

**Affiliations:** Faculty of Medicine, Bar-Ilan University, Safed, Israel

## Abstract

**Introduction:**

Over 1.9 million Arabs live in Israel and constitute 21% of the total population. Despite being a disadvantaged minority population with wide gaps in health indicators, Arabs have higher Human Papillomavirus (HPV) and Measles, Mumps, and Rubella (MMR) vaccination rates compared with the general Jewish population.

**Methods:**

In-depth interviews with 20 health care providers and 30-40 Arab mothers and teens will be conducted to collect information about health system enablers to HPV and MMR vaccination. All interviews are conducted in Arabic by an Arab researcher, audio-recorded, transcribed, and analysed using thematic analysis of the transcripts. Themes will be mapped on the theoretical Social-Ecological Model.

**Results and discussion:**

Preliminary results indicate several health system enablers, such as accessible vaccination services through the school system and mother child clinics, vaccination provided at no cost, and high levels of trust towards healthcare professionals, who are Arab and understand the social and cultural norms of the community. Despite high vaccine uptakes, parents and teens had limited knowledge regarding vaccination, particularly HPV.

**Conclusions:**

This research provides important insights into health system enablers regarding HPV and MMR vaccination among the Arab minority in Israel. Such evidence can serve as a basis for interventions and guidance to improve vaccine uptake among other underserved minority communities in Europe.

